# Flexible trial design in a rare condition

**DOI:** 10.1186/1745-6215-14-S1-P24

**Published:** 2013-11-29

**Authors:** Veronica Moroz, Keith Wheatley, Martin McCabe

**Affiliations:** 1 CRCTU, University of Birmingham, Birmingham, UK; 2 University of Manchester, Manchester, UK; 3 MRC Midland Hub for Trials Methodology Research, University of Birmingham, Birmingham, UK

## 

Running clinical trials in relapsed Ewing sarcoma (ES) is challenging due to its rarity. However randomised clinical trials are essential to evaluate the effectiveness of treatments reliably. Four chemotherapy regimens are currently most often used within Europe for relapsed disease based on small single-arm series with different eligibility criteria, short follow up and variable definitions of outcome measures. There are no comparative data on the relative efficacy, toxicity or acceptability of these regimens. As a result, the evidence base for treatment at relapse is weak and there is widespread variability in chemotherapy delivery and a lack of agreement on which chemotherapy backbone to use as a standard comparator arm for trials of novel agents. We propose a design of a multi-arm multi-stage randomised multicentre international trial to evaluate the efficacy, toxicity and acceptability of these four chemotherapy combinations. Since there is no standard of care in this setting, no standard arm can be defined and the trial will employ a drop-a-loser approach. Initially there will be a 4-way randomisation, with one arm to be dropped after the first stage and a second after the second stage (both based on response rate), with the remaining two arms progressing to a Phase III evaluation based on progression-free survival. We will present the design characteristics and planned analysis methods of this trial in this rare disease.

